# Rapid ART Initiation with BIC/FTC/TAF in People Who Inject Drugs in Greece: Results from a Pilot Single-Arm Study of an Integrated Care Model

**DOI:** 10.3390/microorganisms13122697

**Published:** 2025-11-26

**Authors:** Sotirios Roussos, Konstantinos Protopapas, Elpida Mastrogianni, Charisis Totsikas, Charalampos D. Moschopoulos, Stylianos Bourmpoutelis, Panagiota Resta, Kassandra Procter, Evangelos Kokolesis, Anastasia Antoniadou, Antonios Papadopoulos, Vasileios Papastamopoulos, Apostolos Beloukas, Ioannis Katsarolis, Vana Sypsa, Angelos Hatzakis, Mina Psichogiou

**Affiliations:** 1 Department of Hygiene, Epidemiology and Medical Statistics, Medical School, National and Kapodistrian University of Athens, 11527 Athens, Greecevsipsa@med.uoa.gr (V.S.);; 2 4th Department of Internal Medicine, Attikon University Hospital, Medical School, National and Kapodistrian University of Athens, 12462 Chaidari, Greecebmosxop@yahoo.gr (C.D.M.);; 3 1st Department of Internal Medicine, Laiko General Hospital, National and Kapodistrian University of Athens, 11527 Athens, Greece; elpidamastrogianni@gmail.com (E.M.); sbourmpoutelis@gmail.com (S.B.); 4 5th Department of Internal Medicine-Infectious Diseases Unit, Evangelismos General Hospital, 10676 Athens, Greece; 5 Department of Biomedical Sciences, University of West Attica, 12243 Athens, Greecekprokter@uniwa.gr (K.P.);; 6 National AIDS Reference Centre of Southern Greece, School of Public Health, University of West Attica, 12243 Athens, Greece; 7 Hellenic Scientific Society for the Study of AIDS, Sexually Transmitted and Emerging Diseases, 11475 Athens, Greece; 8 Medical Affairs, Gilead Sciences Hellas, 17564 Paleo Faliro, Greece; ioannis.katsarolis@gilead.com

**Keywords:** rapid ART initiation, people who inject drugs, bictegravir/emtricitabine/tenofovir alafenamide, peer navigation, integrated care model, retention in care, patient-reported outcomes, opioid agonist therapy

## Abstract

People who inject drugs (PWIDs) remain underserved in HIV care. Evidence on rapid antiretroviral therapy (ART) for PWID is limited. We evaluated feasibility, effectiveness, safety, and patient-reported outcomes (PROs) for rapid initiation of bictegravir/emtricitabine/tenofovir alafenamide (BIC/FTC/TAF) supported by a peer navigation in Greece. This is a single-arm, multicenter pilot study including PWIDs (≥18 years) newly diagnosed or relinking after >3 months off ART. Participants started BIC/FTC/TAF on the same day or within 7 days and received peer navigation for 48 weeks. Co-primary endpoints were Week-24 virologic suppression (HIV-1 RNA < 50 copies/mL; FDA Snapshot) and grade 3–4 adverse events (AEs). Secondary endpoints included complete-case suppression at Weeks 24/48, CD4 recovery, retention, and PROs. Outcomes were compared with historical controls from the same centers. Thirty-seven participants were enrolled (83.8% male; median age 33.3 years). Median time to ART was 0 days (vs 78 in controls, *p* < 0.001). Retention was 67.6% at Week 24 and 54.1% at Week 48. In the primary (FDA Snapshot) analysis, suppression was 62.2% and 54.1% at Weeks 24 and 48; in complete-case analyses, results were 92.0% and 100%, respectively. Mean CD4 count increased by 208 cells/μL (95% CI 141–275) at Week 48. Quality of life improved and symptom burden decreased. No grade 3–4 AEs occurred. Rapid BIC/FTC/TAF with peer navigation eliminated delays to ART and achieved favorable virologic, immunologic, and PROs among those retained, with good tolerability. Despite retention challenges, this model appears feasible for PWID and may help close HIV care gaps toward UNAIDS 95–95–95 targets.

## 1. Introduction

People who inject drugs (PWIDs) bear a disproportionate burden of HIV globally, despite major advances in prevention and treatment. While HIV prevalence in the general population is typically less than 1%, prevalence among PWIDs exceeds 20% in regions of Eastern Europe, Asia, and Africa. PWIDs account for approximately one-third of new HIV infections outside Africa [1,2]. Addressing this burden is essential for individual outcomes and for controlling transmission in key populations.

PWIDs with HIV face significant barriers and poorer outcomes across the care continuum. While rapid antiretroviral therapy (ART) initiation has shown promise, evidence of its implementation among PWID remains limited. Previous studies have demonstrated the potential of peer navigation in supporting treatment adherence, but few have evaluated integrated care pathways combining rapid ART initiation with peer support for PWIDs with HIV [3].

The WHO, UNODC, and UNAIDS identify four key HIV-prevention pillars for PWIDs: needle/syringe programs, opioid agonist therapy (OAT), HIV testing and counseling, and ART. Yet structural barriers (stigma, homelessness, criminalization) continue to undermine engagement [2].

Greece illustrates these challenges, having experienced two HIV outbreaks among PWIDs. In Athens, HIV prevalence rose from less than 1% in 2010 to 16.5% in 2013 [4]. During the COVID-19 pandemic, a second outbreak in Thessaloniki increased prevalence among PWIDs from 0.7% in 2018 to 8.7% in 2021 [5]. Despite containment efforts, approximately 90–100 new HIV diagnoses are reported annually [6]. Recent data also indicate concerning trends: treatment coverage remains below 80% and time to ART initiation often exceeds 90 days, with high rates of late presentation (CD4 count <350 cells/μL) [7]. PWIDs have poorer outcomes across the HIV care cascade than other risk groups [8].

Rapid ART initiation (within 0–7 days) reduces loss to follow-up and improves retention [9]. In DIAMOND and FAST, viral suppression reached 82–84% and retention reached 89% at week 48, but PWIDs were scarcely represented, leaving a critical evidence gap [10,11].

Bictegravir/emtricitabine/tenofovir alafenamide (BIC/FTC/TAF) is guideline-recommended for rapid initiation given its efficacy, safety, and high resistance barrier [12,13]. Despite these advantages for PWIDs, who often face inconsistent follow-up, evidence on integrated same-day ART with peer navigation in Europe is lacking.

This pilot study evaluates an integrated care model combining rapid BIC/FTC/TAF initiation with peer navigation among PWIDs with HIV in Greece. In addition to virologic and retention outcomes, we assessed patient-reported measures, recognizing their importance in person-centered care [14]. We hypothesized that this strategy would achieve suppression rates comparable to those in general populations, despite the challenges of engaging PWIDs in continuous care; this study also aimed to inform health policy and improve service delivery in this key population.

## 2. Materials and Methods

### 2.1. Study Design and Setting

We conducted a single-arm, open-label, multicenter pilot study evaluating an integrated care pathway for PWIDs with HIV at three tertiary care centers in Athens, Greece. Enrollment occurred from 22 December 2021 to 12 December 2023, with a pre-specified 48-week follow-up. For the first 24 weeks (primary endpoint window), participants received BIC/FTC/TAF as the study drug. After completing Week 24, participants were offered participation in an open-label extension for an additional 24 weeks (secondary endpoint window) using the commercially available drug (Supplementary Figure S1). The protocol was approved by the Hellenic Scientific Society for the Study of AIDS, Sexually Transmitted and Emerging Diseases, and by the Institutional Review Board at each site; all participants provided written informed consent. The study is registered at ClinicalTrials.gov (identifier NCT07004933).

### 2.2. Sample Size Justification

As a pilot evaluation of a novel integrated care pathway, no formal sample-size calculation was performed. We enrolled 37 participants who received BIC/FTC/TAF with peer navigation support, a size deemed adequate to assess feasibility and obtain preliminary estimates of effectiveness in this hard-to-reach population.

### 2.3. Study Population

We enrolled consecutive, non-selective PWIDs aged ≥ 18 years with documented HIV infection who were either newly diagnosed and ART-naive or relinking after >3 months off ART, with eGFR ≥ 30 mL/min, and willingness to initiate or current participation in OAT; capacity to provide written informed consent. Exclusion criteria included CDC Category C at baseline or suspicion of opportunistic infection precluding rapid ART, severe hepatic impairment, medical conditions compromising safety, contraindicated medications, pregnancy, breastfeeding, or inability to complete study procedures. Full eligibility criteria are provided in the Supplement (Supplementary Table S1).

### 2.4. Historical Control Group

For contextual comparisons, we assembled a historical cohort of PWIDs with HIV who received standard care at the same centers between 2011 and 2022 (n = 174). Controls were adults (≥18 years) with confirmed HIV infection who were either ART-naive or had discontinued ART for >3 months and had an eGFR ≥ 30 mL/min at treatment initiation. During much of this period, ART eligibility followed legacy CD4-based criteria, with universal test-and-treat adopted nationally after 2015. Retention denominators differed between cohorts (diagnosis-to-ART vs. ART start only; Supplementary Figure S2). Additional details on the historical context and the ARISTOTLE program are provided in the Supplement (Supplementary Table S2).

### 2.5. Treatment and Peer Navigation Support

Participants initiated BIC/FTC/TAF within 0–7 days of the first clinic visit. Each participant was assigned a dedicated peer navigator (program field worker with extensive experience in PWID services and health-system navigation) tasked with (1) facilitating linkage to an HIV treatment center within 24 h of enrollment; (2) providing ongoing support throughout the 48-week follow-up; and (3) assisting with appointment scheduling and attendance. This structured support was implemented to address continuity-of-care challenges not routinely covered in Greek HIV care.

### 2.6. Study Assessments and Procedures

Study visits comprised screening, baseline, and follow-up at Weeks 4, 12, 24, 36, and 48. Baseline evaluations included HIV confirmation testing, medical history, physical examination, and laboratory testing. At each follow-up visit, we performed physical examination, laboratory monitoring, assessment of comorbidities and concomitant medications, and safety evaluations. Patient-reported outcomes (PROs) included the EQ-5D-3L, the HIV Symptom Index, the Treatment Satisfaction Questionnaire for Medication (TSQM), and the Simplified Medication Adherence Questionnaire (SMAQ). Details of laboratory panels and PRO scoring are provided in the Supplementary Materials.

### 2.7. Outcome Measures

The primary endpoints were (i) the proportion of participants with plasma HIV-1 RNA < 50 copies/mL at Week 24 (FDA Snapshot analysis), and (ii) the percentage of participants experiencing grade 3 or 4 adverse events (AEs) from baseline through Week 24.

All enrolled participants were included in primary efficacy analyses using an intention-to-treat, missing-equals-failure approach. Participants lost to follow-up or lacking HIV-1 RNA at the assessment time point were classified as not suppressed. We also performed a complete-case analysis among participants retained in care with viral load data at each time point. Virologic failure was defined as HIV-1 RNA ≥ 50 copies/mL at the assessment visit, discontinuation due to lack of efficacy, or missing viral load due to discontinuation/loss to follow-up. Secondary endpoints included viral suppression at Weeks 24 and 48 (<50 copies/mL; missing-excluded), change from baseline in CD4 count and CD4/CD8 ratio; retention in care, treatment discontinuation rates; and resistance development.

Retention definitions differed between cohorts. In the intervention cohort, retention was assessed from baseline (Day 0; same-day ART initiation), capturing all enrolled participants from their first contact with care. In the historical controls, retention was assessed from ART initiation, meaning that individuals who never initiated ART were not included in the denominators. This approach reflects real-world care patterns during the respective periods but could overestimate retention among controls.

### 2.8. Safety Monitoring

Safety was monitored throughout in accordance with the International Council for Harmonisation–Good Clinical Practice. All AEs were recorded and coded. AE severity was graded per the Division of AIDS Table for Grading the Severity of Adult and Pediatric Adverse Events. Details on coding dictionaries, monitoring, and adverse event adjudication are provided in the Supplementary Materials.

### 2.9. Statistical Analysis

We compared outcomes with the historical cohort of PWIDs with HIV treated at the same institutions between 2011 and 2022. Continuous variables are summarized as means with standard deviations (SD) or medians with 25th and 75th percentiles. Categorical variables were presented as counts and percentages. Between-group comparisons used independent-samples *t*-tests or Wilcoxon rank-sum tests for continuous variables and chi-square or Fisher’s exact tests for categorical variables.

Virologic outcomes at Weeks 24 and 48 were evaluated using both missing = failure and complete-case approaches. Repeated measures (CD4 counts, CD4/CD8 ratios, and PROs) were analyzed using linear mixed-effects models with random intercepts per participant, and visit was modeled as a categorical fixed effect. Model specifications are provided in Supplementary Tables S3–S6.

All tests were two-sided with α = 0.05. Analyses were conducted in Stata (Release 19.5) [15].

## 3. Results

### 3.1. Study Population and Baseline Characteristics

Between December 2021 and December 2023, 37 participants were enrolled. The cohort was predominantly male (31/37, 83.8%) with a median age of 33.3 years (25th–75th percentile: 31.8–42.6). Most participants were white (36/37, 97.3%) and had completed basic education (22/36 [61.1%]). Thirty-three participants (91.7%) were unemployed, 26 (70.3%) were single, and 4 (10.8%) reported housing instability. Nine participants (24.3%) were enrolled in OAT at baseline (Table 1).

### 3.2. Retention

**Figure 1:**
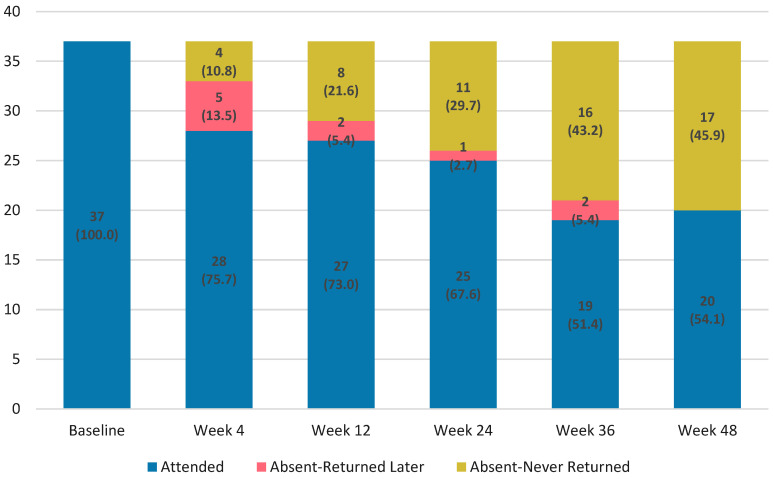
Retention and attendance patterns over 48 weeks following rapid BIC/FTC/TAF initiation among people who inject drugs.

Retention was lower than in controls at Week 24 (67.6% vs. 86.7%; *p* = 0.005) and Week 48 (54.1% vs. 80.9%; *p* < 0.001). Note that denominators differ between cohorts (intervention measured from diagnosis/ART start; controls from ART initiation; Supplementary Figure S2). Participant retention declined progressively over 48 weeks (Figure 1). Visit deviations rose from 1 day in Week 4 to 29 days in Week 48, showing greater scheduling variability (Supplementary Figure S3). Of 37 enrolled, 25 (67.6%) were retained at Week 24 and 20 (54.1%) completed Week 48. Permanent discontinuation occurred in 4 (10.8%) by Week 4 and 17 (45.9%) by Week 48. Some participants missed interim visits but later re-engaged, whereas others permanently discontinued after a missed visit (Figure 1, Supplementary Figure S4).

### 3.3. Time to ART Initiation

In our integrated care model, the median time (25th–75th percentile) from first visit to ART initiation was 0 days (0–0) compared with 78 days (26–274) in historical controls (*p* < 0.001). Compared with historical practice, same-day ART eliminated approximately 60,000 pre-ART viremia days, a period otherwise associated with high transmission risk (Supplementary Figure S2).

### 3.4. Virologic Outcomes

**Figure 2:**
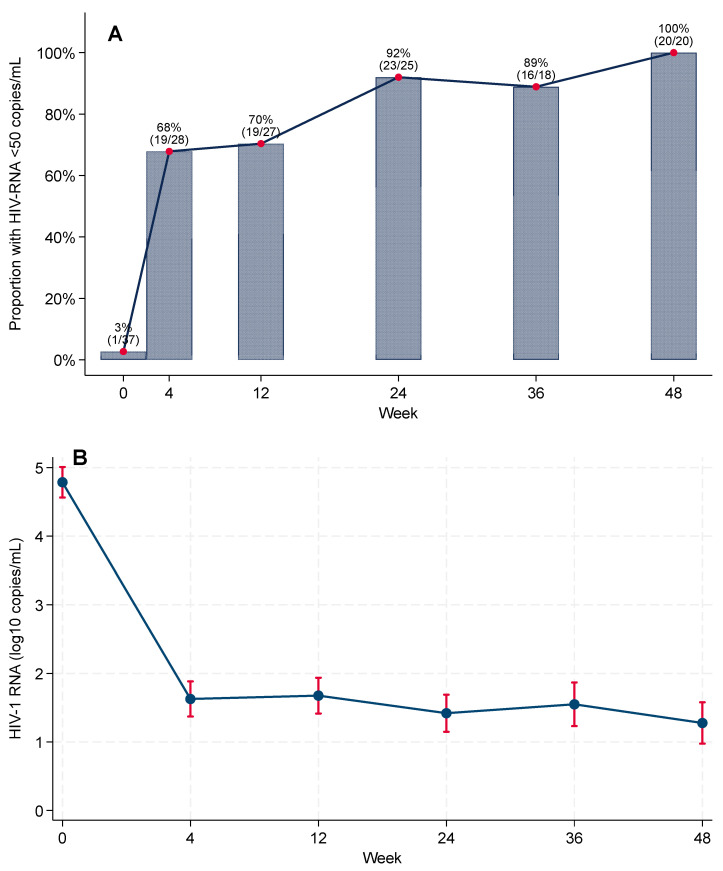
Virologic outcomes during 48-week follow-up after rapid ART initiation with BIC/FTC/TAF. (**A**) Proportion with HIV-1 RNA < 50 copies/mL among participants with viral load assessed at each visit (complete-case). Numbers above bars show suppressed/assessed. (**B**) Cohort-level mean log10 HIV-1 RNA over time from mixed-effects models with 95% CIs.

In the complete-case analysis, virologic suppression (HIV-1 RNA < 50 copies/mL) increased rapidly after BIC/FTC/TAF initiation: 1/37 (2.7%) at baseline, 19/28 (67.9%) at Week 4, 19/27 (70.4%) at Week 12, 23/25 (92.0%) at Week 24, 16/18 (88.9%) at Week 36, and 20/20 (100%) at Week 48 (Figure 2; Supplementary Table S3).

In the FDA Snapshot (primary) analysis, virologic suppression was 62.2% at Week 24 (23/37) and 54.1% at Week 48 (20/37), whereas in complete-case (observed) analyses, it was 92.0% and 100.0%, respectively (Supplementary Tables S3 and S4). No discontinuations were attributed to confirmed virologic failure. Among snapshot failures, 2/37 (5.4%) had HIV-1 RNA ≥ 50 copies/mL at Week 24 (declining to 0/37 at Week 48); the remainder were due to loss to follow-up or discontinuation for reasons unrelated to efficacy (12/37 [32.4%] at Week 24; 17/37 [45.9%] at Week 48).

Genotypic resistance testing was performed at baseline in 35/37 participants (94.6%). No baseline resistance to BIC/FTC/TAF components was identified. During follow-up, only one participant underwent additional resistance testing (at Week 36), with no evidence of emergent resistance. No treatment modifications were attributed to baseline or acquired resistance.

Baseline viremia was higher in the integrated-care cohort than in historical controls, with median (25th–75th percentile) log10 HIV-1 RNA of 5.07 (4.32–5.36) vs. 4.49 (3.80–4.97) in controls (*p* = 0.022). Using a missing-equals-excluded analysis, suppression was significantly higher at Week 24 in the integrated model (92.0% vs. 61.8%, *p* = 0.006) and numerically higher at Week 48 (100% vs. 84.5%, *p* = 0.068).

As shown in Figure 2B, cohort-level HIV-1 RNA declined after ART initiation, with an overall statistically significant reduction in model-based mean viral load across the study period (global test for the time effect in linear mixed-effects models, *p* < 0.001).

Virologic rebound (≥50 copies/mL after prior suppression) occurred in 6/37 (16.2%): 4/6 (66.7%) at Week 12, 1/6 (16.7%) at Week 24, and 1/6 (16.7%) at Week 36 (Supplementary Figure S5). Most participants (31/37, 83.8%) maintained suppression once achieved.

### 3.5. Immunologic Response

**Figure 3:**
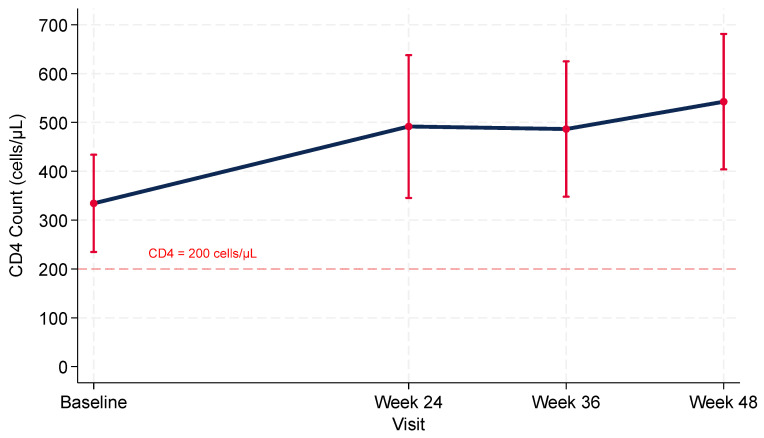
CD4 + T-Cell Trajectories after Rapid Bictegravir/Emtricitabine/Tenofovir Alafenamide Initiation among PWIDs with HIV: Model-based Means with 95% Confidence Intervals.

Mean baseline CD4 count was 334 cells/μL (95% CI, 231–438). In linear mixed-effects models, mean CD4 count increased by 159 cells/μL (95% CI, 97–221; *p* < 0.001) at Week 24, 187 cells/μL (95% CI, 117–256; *p* < 0.001) at Week 36, and 208 cells/μL (95% CI, 141–275; *p* < 0.001) at Week 48 (Figure 3). Substantial between-participant heterogeneity in CD4 count trajectories was observed (*p* < 0.001). By Week 48, 90.0% (95% CI, 68.3–98.8%) had CD4 count >200 cells/μL. The CD4/CD8 ratio improved significantly over time, from a mean of 0.38 at baseline to 0.71 at Week 48 (mixed-model estimate +0.29, 95% CI 0.18–0.40; *p* < 0.001) (Supplementary Table S6).

### 3.6. Health-Related Quality of Life

In mixed-effects regression with robust standard errors, there was a significant overall time effect on EQ-5D-3L utility (global test of visit indicators: *p* = 0.004). Compared with baseline, only Week 36 (visit 4) was higher (β = 0.113; 95% CI 0.055–0.172; *p* < 0.001).

Self-reported health status on the EQ-5D visual analog scale demonstrated a consistent progressive improvement from baseline (mean 46.7, SD 20.7) to Week 4 (52.1, 22.2), Week 12 (55.2, 21.4), Week 24 (55.6, 23.5), Week 36 (61.8, 23.0), and Week 48 (67.4, 20.4).

### 3.7. HIV Symptom Burden

At baseline, participants reported a mean of 6.8 (SD 3.9) symptoms causing at least moderate bother (HIV Symptom Index ≥ 3); the median was 6.5 (25th–75th percentile 4–9; range 0–18).

**Figure 4:**
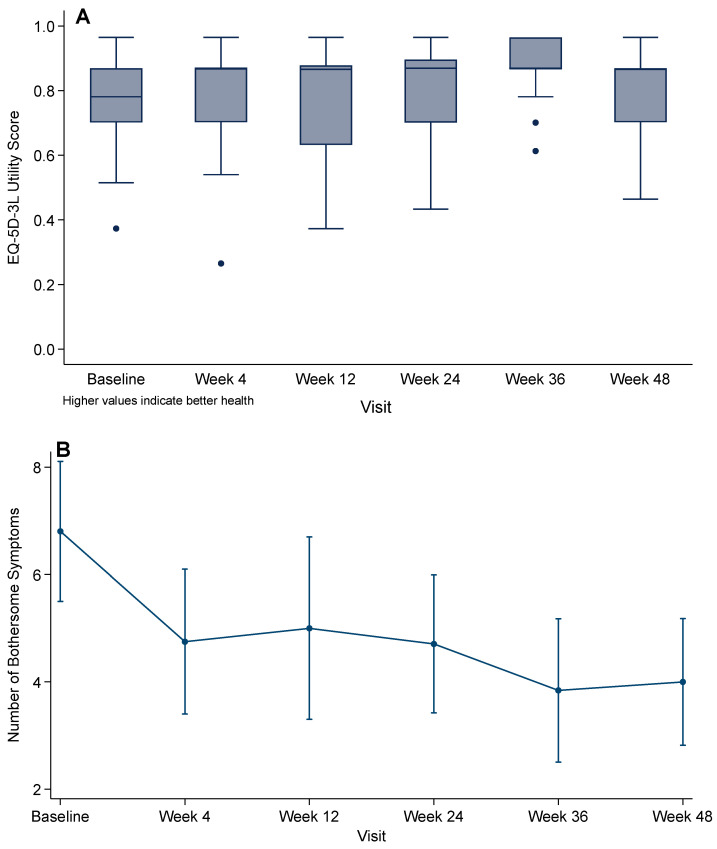
Patient-reported outcomes over 48 weeks after rapid BIC/FTC/TAF initiation: (**A**) EQ-5D-3L utility score (UK value set); (**B**) HIV Symptom Index (mean number of moderately or more bothersome symptoms). Points show means with 95% CIs; *p*-values from linear mixed-effects models compare each visit with baseline.

Symptom burden decreased over time (Figure 4): mean bothersome symptoms fell to 4.8 (SD 3.5) at Week 4, 5.0 (4.3) at Week 12, 4.7 (3.0) at Week 24, 3.8 (2.8) at Week 36, and 4.0 (2.4) at Week 48. This represents a mean reduction of 4.1 symptoms (SD 4.9) from baseline to Week 48 (*p* < 0.001, paired *t*-test). Mixed-effects models confirmed significant reductions versus baseline at Week 4 (*p* = 0.007), Week 12 (*p* = 0.012), Week 24 (*p* = 0.001), and Weeks 36 and 48 (both *p* < 0.001).

### 3.8. Medication Adherence

Self-reported perfect adherence (SMAQ = 6) was 63.0% (17/27) at Week 4, 44.4% (12/27) at Week 12, 44.0% (11/25) at Week 24, 52.6% (10/19) at Week 36, and 57.9% (11/19) at Week 48. Sustained perfect adherence between consecutive visits was uncommon: 30.4% (7/23) maintained perfect adherence from Week 4 to 12, 25.0% (6/24) from Week 12 to 24, 33.3% (6/18) from Week 24 to 36, and 47.1% (8/17) from Week 36 to 48. No consistent association with viral suppression was observed. All participants seen at Week 48 (n = 19) were suppressed irrespective of SMAQ status.

### 3.9. Safety

BIC/FTC/TAF demonstrated good tolerability. Six participants (16.2%) reported adverse events during the study, with no grade 3 or 4 adverse events recorded. Clinical laboratory assessments (lipids, hematology, renal/hepatic chemistry, urinalysis, coagulation) showed no consistent or clinically meaningful abnormalities; among evaluable assessments, no clinically significant lipid abnormalities were flagged and median changes from baseline were small. Treatment satisfaction remained high and stable across visits (TSQM global item; median: 7, 25th–75th percentile: 6–7; observed).

## 4. Discussion

This pilot study evaluated an integrated care model combining rapid initiation of BIC/FTC/TAF with peer navigation support for PWIDs with HIV in Greece. Same-day ART initiation proved feasible in this highly vulnerable group, achieving high virologic and immunologic responses among participants retained in care (n = 20 at Week 48), with favorable patient-reported outcomes and an acceptable safety profile. Declining retention has limited population impact, highlighting the need for strategies to sustain engagement.

### 4.1. Interpretation of Findings

This study adds early European evidence for an integrated model combining same-day BIC/FTC/TAF initiation with structured peer navigation among PWIDs, a population systematically underrepresented in trials despite bearing a disproportionate HIV burden. Time from diagnosis to treatment fell dramatically, from 78 days in historical controls to same-day initiation, eliminating the common pre-ART dropout window. Our findings extend DIAMOND and FAST studies [10,11], showing similar gains in PWIDs with adequate support.

Virologic outcomes were favorable. Complete-case suppression at Week 48 reached 100% (n = 20), despite higher baseline viremia than historical controls. As prespecified, the FDA Snapshot analysis provides the population-level measure, because loss to follow-up counts as virologic failure in this framework. Snapshot suppression was 62.2% at Week 24 and 54.1% at Week 48, indicating that limited retention—not virologic performance of BIC/FTC/TAF—is the primary barrier to achieving population-level viral suppression. No emergent resistance was detected, consistent with the high genetic barrier of BIC/FTC/TAF and its capacity to maintain suppression even with imperfect adherence.

Rapid viral decline was accompanied by improved patient-reported outcomes, including reduced symptom burden, higher EQ-5D scores, and better quality of life. These early gains may reinforce engagement through a positive feedback loop between symptom relief and adherence. Immunologic recovery was robust, with a mean CD4 gain of 208 cells/µL by Week 48 and an increase in the CD4/CD8 ratio from 0.38 at baseline to 0.67, comparable to responses reported in non-PWID populations.

### 4.2. Retention Challenges

Retention was the principal limitation: 54% completed Week 48 vs. 81% in historical controls. Lower baseline enrollment in OAT (24% vs. 42% in controls) likely contributed to reduced retention. Additional factors included housing instability, unemployment, competing priorities, and COVID-19 disruptions. Visit timing drifted from a median deviation of 1 day at Week 4 to 29 days at Week 48, underscoring the mismatch between rigid schedules and PWID engagement. Comparisons with historical controls should be interpreted cautiously, as many were linked during the ARISTOTLE seek–test–treat initiative integrated with OAT, which likely enhanced retention and favored control outcomes (see Supplementary Materials). Overall, while rapid initiation prevents early attrition, sustaining retention requires flexible, durable strategies.

### 4.3. Implications for the UNAIDS 95-95-95 Targets

Our findings are directly relevant to the UNAIDS 95-95-95 goals. While all retained participants achieved suppression at Week 48, the WHO definition of the third “95” applies to all people living with HIV, not just those who are retained. With nearly half lost to follow-up, population-level suppression likely fell short. Nevertheless, compared with historical controls (58.8% at Week 24; 82.9% at Week 48), our model substantially narrowed existing gaps, highlighting its potential to accelerate progress toward global targets.

### 4.4. Implementation Across Settings

Beyond Greece, this pragmatic Test–Start–Support model is particularly relevant in Eastern Europe and Central Asia, where PWIDs drive a substantial share of new infections, as it integrates same-day ART with OAT linkage, harm-reduction services, and peer navigation within diverse health systems. Its core elements—same-day ART initiation, simplified baseline assessments, and structured peer navigation—are also adaptable to resource-limited or fragmented healthcare settings through partnerships with community-based organizations, mobile clinics, and low-threshold harm-reduction services.

Evidence from Spain and the United States shows that rapid-start pathways combined with peer or case-management support can achieve retention of 60–80% and virologic suppression above 90% even in socially unstable populations. Together, these findings indicate that rapid initiation with a potent single-tablet regimen and peer navigation is scalable across health systems. The Test–Start–Support (TSS) strategy, Test (same-day diagnosis), Start (immediate ART before leaving the clinic), Support (ongoing peer navigation), offers a pragmatic framework. Even partial adoption, such as same-day initiation or peer support alone, may yield better outcomes than standard care.

Recent studies support this approach. Ryan et al. [16] implemented same-day BIC/FTC/TAF initiation in Spain with mobile outreach and nonjudgmental counseling, achieving 64.4% retention and 96.9% virologic suppression at Week 48. HIV acquisition through injection drug use was associated with lower retention, highlighting the added value of peer navigation. Similarly, the COMEBACK study in the U.S. Reference [17] combined rapid re-initiation of BIC/FTC/TAF with case management addressing social determinants, achieving 79% suppression among engaged participants at Week 48. Together, these findings indicate that rapid initiation strategies can succeed across diverse settings when supported by structural and peer-based interventions.

### 4.5. Policy and Practice Recommendations

Effective implementation hinges on integrating HIV and substance use services, co-locating clinics with OAT programs, and using shared records. HIV centers should adopt rapid, PWID-focused ART protocols with streamlined baseline assessments enabling same-day initiation. Peer navigator programs require stable funding, standardized training, and formal roles within care teams. Service delivery must be flexible, including extended hours, walk-in visits, telehealth, and community outreach, to accommodate irregular engagement. High-barrier regimens such as BIC/FTC/TAF should be prioritized given resilience to adherence lapses. Routine follow-up should incorporate patient-reported outcomes, and retention requires case management, transportation, or material support, and scale-up of underutilized OAT programs.

### 4.6. Limitations

This single-arm pilot with modest sample size limits generalizability and precludes subgroup analyses. Comparisons with historical controls are exploratory and subject to calendar-time and contextual confounding, including pandemic-related disruptions and service-environment differences. Importantly, historical controls were linked to care within a substantially different service environment, including periods of intensified community-based interventions such as ARISTOTLE, which may have facilitated linkage or retention relative to routine care. National ART guidelines also changed substantially between 2011 and 2022 (transition from CD4-based eligibility to universal test-and-treat), affecting baseline characteristics and timing of ART initiation in the historical cohort. Additionally, retention estimates are not directly comparable because denominators differed between cohorts (diagnosis-to-ART in the intervention cohort vs. ART start in historical controls; Supplementary Figure S2). Finally, our findings reflect Greece’s universal healthcare system and may not fully generalize to settings with different resources or service organization.

### 4.7. Future Directions

These recommendations are informed by barriers directly observed in our cohort, including low baseline OAT enrollment, housing instability, and progressive visit drift over time, all of which were associated with early disengagement. As such, the strategies proposed below are designed to target these structural drivers of poor retention. Future implementation research should evaluate targeted strategies to improve retention among PWIDs, including co-location of HIV and OAT services, walk-in or low-threshold ART refill points, peer-led re-engagement protocols, outreach-based follow-up for missed visits, transportation assistance, and structured case management addressing housing instability, unemployment, bureaucratic barriers, and mental health needs.

Further research should evaluate the durability of responses and test interventions to enhance retention. Multicenter or randomized studies could help isolate effective components and assess cost-effectiveness. Greater attention to substance use, mental health, and social determinants will also be essential. Economic evaluations are needed to inform policymakers on the value of this approach compared with standard care, particularly in resource-limited settings.

## 5. Conclusions

Rapid initiation of BIC/FTC/TAF combined with peer navigation proved feasible, effective, and safe among PWIDs with HIV in Greece, delivering high virologic and immunologic responses among retained participants and improving quality of life. Although retention in care remains the central challenge, this integrated model addresses long-standing barriers to ART initiation and offers a pragmatic framework for improving outcomes in a marginalized population. By advancing progress toward global UNAIDS targets, the Test–Start–Support strategy represents a blueprint for equitable HIV care delivery for PWIDs and merits further evaluation in larger and more diverse settings.

## Figures and Tables

**Table 1:** Baseline Demographic and Clinical Characteristics.

Characteristic	Cases
Demographics	
Female sex, n (%)	6 (16.2)
Age, years, median (25th–75th percentile)	33.3 (31.8–42.6)
Race, n (%)	
White	36 (97.3)
Other	1 (2.7)
Socioeconomic Status	
Education level, n (%)	
Low/Basic	22 (61.1)
Medium	10 (27.8)
High	4 (11.1)
Employment Status, n (%)	
Full-Time Employee	1 (2.8)
Part-Time Employee	2 (5.6)
Unemployed	33 (91.7)
Family Status, n (%)	
Married	3 (8.1)
In a relationship	3 (8.1)
Single	26 (70.3)
Divorced	5 (13.5)
Housing instability, n (%)	4 (10.8)
Clinical Characteristics	
In OAT Program Status, yes n (%)	9 (24.3)
Hospitalized at baseline, yes n (%)	4 (11.4)

OAT = Opioid Agonist Therapy.

## Data Availability

The datasets presented in this article are not readily available because the data contain sensitive information and participant confidentiality must be protected. Requests to access the datasets should be directed to the corresponding author.

## Supplementary Materials

The following supporting information can be downloaded at https://www.mdpi.com/article/10.3390/microorganisms13122697/s1. Table S1: Eligibility Criteria for Study Enrollment. Table S2: Baseline Demographic and Clinical Characteristics. Table S3: Virologic Suppression by Complete-Case Analysis. Table S4: Virologic outcomes by FDA Snapshot at Weeks 24 and 48 (missing=failure). Table S5: CD4+ T-Cell Recovery Over 48 Weeks. Table S6: CD4/CD8 Ratio Trajectories. Estimates represent change from baseline (mixed-effects model). Figure S1: CONSORT-Style Study Schema. Figure S2: Definition of Week 24 in Intervention vs. Historical Controls. Figure S3: Visit Adherence Over Time. Figure S4: Visit completion patterns from baseline through Week 48. Figure S5: Individual Viral Load Trajectories.

## References

[1] Feelemyer J., Des Jarlais D., Arasteh K., Uuskula A. (2015). Adherence to antiretroviral medications among persons who inject drugs in transitional, low and middle income countries: An international systematic review. AIDS Behav..

[2] United Nations Office on Drugs and Crime-UNODC Drug Use and HIV. https://www.unodc.org/unodc/en/hiv-aids/new/drug-use_and_HIV.html.

[3] Uuskula A., Feelemyer J., Des Jarlais D.C. (2023). HIV treatment, antiretroviral adherence and AIDS mortality in people who inject drugs: A scoping review. Eur. J. Public Health.

[4] Sypsa V., Psichogiou M., Paraskevis D., Nikolopoulos G., Tsiara C., Paraskeva D., Micha K., Malliori M., Pharris A., Wiessing L. (2017). Rapid Decline in HIV Incidence Among Persons Who Inject Drugs During a Fast-Track Combination Prevention Program After an HIV Outbreak in Athens. J. Infect. Dis..

[5] Sypsa V., Roussos S., Tsirogianni E., Tsiara C., Paraskeva D., Chrysanthidis T., Chatzidimitriou D., Papadimitriou E., Paraskevis D., Goulis I. (2023). A new outbreak of HIV infection among people who inject drugs during the COVID-19 pandemic in Greece. Int. J. Drug Policy.

[6] National Public Health Organization (2023). HIV/AIDS Surveillance Report in Greece, 31-12-2023 (Issue 38).

[7] Tsiara C. The course of the epidemic among PWID and other populations. Proceedings of the 8th Panhellenic Meeting for AIDS and hepatitis.

[8] Vourli G., Noori T., Pharris A., Porter K., Axelsson M., Begovac J., Cazein F., Costagliola D., Cowan S., Croxford S. (2020). Human Immunodeficiency Virus Continuum of Care in 11 European Union Countries at the End of 2016 Overall and by Key Population: Have We Made Progress?. Clin. Infect. Dis..

[9] World Health Organization Guidelines for Managing Advanced HIV Disease and Rapid Initiation of Antiretroviral Therapy. https://www.ncbi.nlm.nih.gov/books/NBK475972/.

[10] Huhn G.D., Crofoot G., Ramgopal M., Gathe J., Bolan R., Luo D., Simonson R.B., Nettles R.E., Benson C., Dunn K. (2020). Darunavir/Cobicistat/Emtricitabine/Tenofovir Alafenamide in a Rapid-Initiation Model of Care for Human Immunodeficiency Virus Type 1 Infection: Primary Analysis of the DIAMOND Study. Clin. Infect. Dis..

[11] Bachelard A., Isernia V., Charpentier C., Benalycherif A., Mora M., Donadille C., Duvivier C., Lacombe K., El Mouhebb M., Spire B. (2023). Same-day initiation of bictegravir/emtricitabine/tenofovir alafenamide: Week 48 results of the FAST study-IMEA 055. J. Antimicrob. Chemother..

[12] Deeks E.D. (2018). Bictegravir/Emtricitabine/Tenofovir Alafenamide: A Review in HIV-1 Infection. Drugs.

[13] Gallant J., Lazzarin A., Mills A., Orkin C., Podzamczer D., Tebas P., Girard P.M., Brar I., Daar E.S., Wohl D. (2017). Bictegravir, emtricitabine, and tenofovir alafenamide versus dolutegravir, abacavir, and lamivudine for initial treatment of HIV-1 infection (GS-US-380-1489): A double-blind, multicentre, phase 3, randomised controlled non-inferiority trial. Lancet.

[14] Simpson K.N., Hanson K.A., Harding G., Haider S., Tawadrous M., Khachatryan A., Pashos C.L., Wu A.W. (2013). Patient reported outcome instruments used in clinical trials of HIV-infected adults on NNRTI-based therapy: A 10-year review. Health Qual. Life Outcomes.

[15] StataCorp (2025). Stata Statistical Software: Release 19.5.

[16] Ryan P., Cuevas G., Torres P., Laguna L., Brazal B., Miguel M.D., Matarranz M., Manzano S., Torres-Macho J., Martin-Gonzalez L. Simplified Access and Retention Model for Vulnerable People With HIV: SIMPLIFIED Study Results. Proceedings of the Conference on Retroviruses and Opportunistic Infections.

[17] Burke K., Roden L., Keckler K., DeMarco C., Cortez Valadez P., Grennan D., Osborn R., Huhn G. Social Determinants of Health (SDoH) impact on Viral Suppression (VS) in a 48-week Low Barrier Care (LBC) study for rapid Antiretroviral Therapy (ART) reinitiation among persons with HIV (PWH) lost-to-care. Proceedings of the 25th International AIDS Conference.

