# Supplementary material for: Rapid ART Initiation with BIC/FTC/TAF in People Who Inject Drugs in Greece: Results from a Pilot Single-Arm Study of an Integrated Care Model
Source: Microorganisms. 2025 Nov 26;13(12):2697. doi: 10.3390/microorganisms13122697 (PMC12735862; doi:10.3390/microorganisms13122697)

microorganisms

MDPI

SUPPLEMENTARY APPENDIX

Methods

Eligibility criteria are summarized in Supplementary Table S1, which lists the full inclusion and exclusion requirements applied at screening.

Supplementary Table S1. Eligibility Criteria for Study Enrollment

Summary of inclusion and exclusion criteria applied at screening to determine participant eligibility.

Inclusion Criteria	Exclusion Criteria
Age ≥18 years	CDC Category C at baseline or suspicion of opportunistic infection precluding rapid ART per 2020 EACS guidelines
Documented HIV-1 infection	ALT >5× upper limit of normal or Child–Pugh >9
ART-naïve or off ART for >3 months	Severe medical conditions compromising safety, or conditions impairing drug absorption
eGFR ≥30 mL/min	Ongoing treatment with contraindicated medications within 14 days prior to enrollment
Willingness to initiate or continue opioid agonist therapy (OAT)	Pregnancy or breastfeeding
Ability to provide informed consent	Participation in another interventional clinical trial
	Inability to comply with study procedures as judged by the investigator

Abbreviations: ART, antiretroviral therapy; eGFR, estimated glomerular filtration rate; OAT, opioid agonist therapy.

Visit Schedule and Assessments

Participants underwent screening (Day −7 to −1), baseline (Day 0), and follow-up visits at weeks 4, 12, 24, 36, and 48. At baseline, ART was initiated following confirmation of HIV infection and eligibility assessment. Follow-up visits included physical examination, HIV-1 RNA, CD4/CD8 counts, and safety laboratory testing. Laboratory panels included hematology, chemistry, lipid profile, urinalysis, and coagulation parameters. Patient-reported outcomes (PROs) were assessed at scheduled visits using the EQ-5D-3L, HIV Symptom Index (HIV-SI), Treatment Satisfaction Questionnaire for Medication (TSQM), and the Simplified Medication Adherence Questionnaire (SMAQ).

Safety Monitoring

All adverse events (AEs) were documented and coded using MedDRA version 24.0, with concomitant medications coded using the WHO Drug Dictionary. Severity of AEs was graded according to the DAIDS Table for Grading the Severity of Adult and Pediatric Adverse Events, Version 2.1. On-site monitoring was performed throughout the study to ensure protocol adherence and data integrity.

https://doi.org/10.3390/microorganisms13122697

Historical Control Cohort

Historical controls were PWID with HIV who received care at the same institutions between 2011 and 2022. This period overlapped the post-outbreak phase following the 2012 PWID HIV epidemic in Athens. ART eligibility followed national guidelines at the time: CD4 <350 cells/μL until 2015, after which a universal test-and-treat policy was adopted. Many individuals were linked to care during or shortly after the ARISTOTLE seek-test-treat initiative (2012–2013), which integrated HIV testing, counseling, and opioid agonist therapy. During this period, some historical controls may have accessed more comprehensive, integrated services than those typically available during the present study period. Eligible controls were ≥18 years, ART-naïve or off ART for >3 months, and had an eGFR ≥30 mL/min at treatment initiation. Controls were included consecutively and were unmatched. Follow-up time points for historical controls were defined relative to ART initiation, whereas in the intervention cohort baseline coincided with same-day ART (Day 0); thus, retention denominators differ (diagnosis-to-ART vs ART start). Because control data were abstracted retrospectively from treating physicians/medical records rather than a centralized registry, individuals who linked to care but never initiated ART, or initiated and did not return, may have been under-ascertained, potentially overestimating retention in controls.

Statistical Analysis – Technical Details

Longitudinal changes in CD4 counts, CD4/CD8 ratios, and patient-reported outcomes (PROs) were analyzed using linear mixed-effects models with random intercepts per participant and visit was modeled as a categorical fixed effect. An unstructured covariance matrix was assumed for within-participant errors, and robust (Huber–White) standard errors were applied to account for potential heteroscedasticity and mild model misspecification. Global F-tests were used to assess overall time effects. Virologic outcomes were analyzed using both FDA Snapshot (missing = failure) and complete-case (missing excluded) approaches.

Abbreviations used in the supplementary tables and figures are listed below for clarity.

**Abbreviation**	**Definition**
AE	adverse event
ART	antiretroviral therapy
CI	confidence interval
DAIDS	Division of AIDS (NIH)
EACS	European AIDS Clinical Society
eGFR	estimated glomerular filtration rate
FDA (Snapshot)	U.S. Food and Drug Administration algorithm classifying missing HIV-1 RNA as failure
GCP	Good Clinical Practice
IQR	interquartile range
MedDRA	Medical Dictionary for Regulatory Activities
OAT	opioid agonist therapy
PROs	patient-reported outcomes
SMAQ	Simplified Medication Adherence Questionnaire
TSQM	Treatment Satisfaction Questionnaire for Medication
VL	viral load (HIV-1 RNA)
WHO-DD	World Health Organization Drug Dictionary

Note: We use VL and HIV-1 RNA synonymously throughout.

Results

Comparison of baseline characteristics between HIV patients in rapid-initiation versus standard-care groups

To evaluate potential differences between treatment approaches, we compared baseline demographic and clinical characteristics between cases (n = 37) receiving rapid ART initiation and historical controls (n = 174) receiving standard care. As shown in Table S2, the demographic profiles were similar between groups. The proportion of female participants (16.2% vs. 16.1%, p = 0.985) and median age at diagnosis (33.3 vs. 35.0 years, p = 0.387) were comparable. Socioeconomic factors, specifically the lack of stable housing, were not significantly different between cases and controls (10.8% vs. 10.5%, p = 0.959). However, a significantly lower proportion of cases were enrolled in opioid agonist therapy programs compared to controls (24.3% vs. 41.9%, p = 0.047), likely reflecting temporal variation in OAT enrollment and service availability between the groups (Supplementary Table S2).

Supplementary Table S2. Baseline Demographic and Clinical Characteristics

Comparison of baseline characteristics between rapid-initiation participants and historical controls.

Characteristic	Cases (N=37)	Historical Controls (N=174)	p-value
Demographics			
Female sex, n (%)	6 (16.2)	28 (16.1)	0.985[1]
Age at diagnosis, years, median (25th–75th percentile)	33.3 (31.8–42.6)	35.0 (30.4–39.1)	0.387[2]
Socioeconomic Status			
Home/shelter availability, no n (%)	4 (10.8)	18 (10.5)	0.959[1]
Clinical Characteristics			
In OAT Program Status, yes n (%)	9 (24.3)	72 (41.9)	0.047[1]

[1] Pearson's chi-square test, [2] Kruskal-Wallis's test. Abbreviations: OAT = Opioid Agonist Therapy

The timing of study visits showed increasing variability from the scheduled protocol throughout the follow-up period (Supplementary Figure S2). Median time deviations from scheduled visits were modest at Week 4 (1 day, 25th–75th percentile: 0–2) but progressively increased with study duration: 9 days (25th–75th percentile: 6–21) at Week 12, 15 days (25th–75th percentile: 8–22) at Week 24, 27 days (25th–75th percentile: 20–29) at Week 36, and 29 days (25th–75th percentile: 24–35) at Week 48. This pattern of increasing time deviations at later visits is consistent with the challenges of maintaining strict appointment schedules in this vulnerable population. Despite these deviations, the majority of participants who remained in the study completed their visits within the predefined acceptable windows, allowing for valid assessment of study endpoints according to the protocol.

Supplementary Table S3. Virologic Suppression by Complete-Case Analysis

Proportion of participants achieving HIV-1 RNA <50 copies/mL at each visit; observed data only (complete cases).

Time (week)	Assessed N	HIV-1 RNA (<50 copies/mL) N (%)
0	37	1 (2.7)
4	28	19 (67.9)
12	27	19 (70.4)
24	25	23 (92.0)
36	18	16 (88.9)
48	20	20 (100.0)

Supplementary Table S4. Virologic outcomes by FDA Snapshot at Weeks 24 and 48 (missing=failure).

All enrolled participants are included at each visit; missing HIV-1 RNA is classified as not suppressed. Rows show Snapshot components (suppressed; ≥50 copies/mL; discontinued for poor efficacy; discontinued for other reasons with last VL ≥50 or <50; missing on drug). For reference, complete-case suppression (observed data only among those assessed at the visit) is shown in the last row.

	24 weeks	48 weeks
**Virologic Success**		
HIV-1 RNA <50 copies/mL (in window)	23	20
**Virologic Failure**		
HIV-1 RNA ≥50 copies/mL (in window)	2	0
Discontinued due to lack of efficacy	0	0
Discontinued because of adverse event or death	0	0
Discontinued for other reasons, last HIV-1 RNA ≥50 copies/mL	8	9
Discontinued for other reasons, last HIV-1 RNA <50 copies/mL	3	8
No virologic data in window but on study drug	1	0
**Summary**		
HIV-1 RNA <50 copies/mL , FDA Snapshot (n/N, %)	23/37 (62%)	20/37 (54%)
HIV-1 RNA <50 copies/mL , Observed (complete-case; n/N, %)	23/25 (92%)	20/20 (100%)

Supplementary Table S5. CD4+ T-Cell Recovery Over 48 Weeks

Mean CD4+ T-cell counts and changes from baseline, estimated with linear mixed-effects models (95% confidence intervals).

Time point	Mean CD4+ count (cells/μL)	Mean change from baseline (cells/μL)	95% CI	p-value
Baseline	334 (95% CI: 231, 438)	,	,	,
Week 24	493	159	97 to 221	< 0.001
Week 36	521	187	117 to 256	< 0.001
Week 48	542	208	141 to 275	< 0.001

CI = confidence interval.

Supplementary Table S6. CD4/CD8 Ratio Trajectories. Estimates represent change from baseline (mixed-effects model).

CD4/CD8 ratio estimates from baseline through Week 48, derived from mixed-effects models (95% confidence intervals).

Time (week)	Estimate (change from baseline)	95% CI	p-value
Baseline (Week 0)	0.38	0.28 – 0.48	<0.001
Week 24	+0.19	0.08 – 0.31	0.001
Week 36	+0.22	0.12 – 0.32	<0.001
Week 48	+0.29	0.18 – 0.40	<0.001

CI = confidence interval.

Supplementary Figure S1. CONSORT-Style Study Schema

Overview of screening, baseline (same-day ART), and follow-up visits at Weeks 4, 12, 24, 36, and 48; shows assessment windows and core procedures (VL, CD4/CD8, safety labs, PROs).

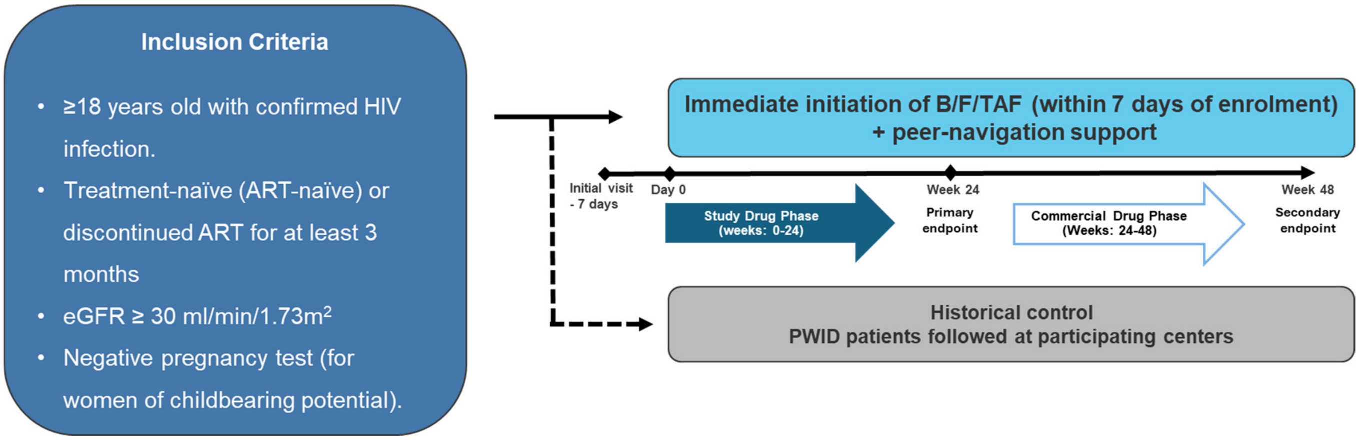

Supplementary Figure S2. Definition of Week 24 in Intervention vs. Historical Controls

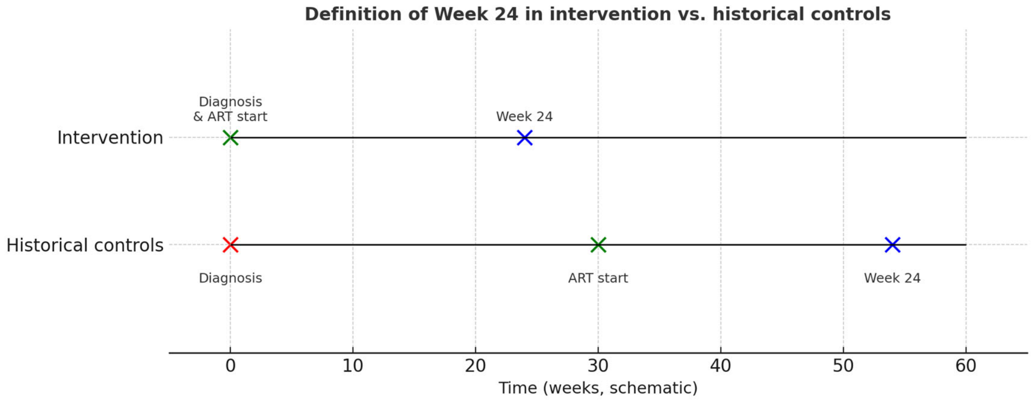

Schematic illustrating assessment timing: in the intervention cohort, Week 24 was counted from ART start (same-day as diagnosis), whereas in historical controls it was counted from ART initiation, which often followed a delay after diagnosis. This difference affects retention denominators and inflates pre-ART viremia-days in controls.

For the historical control group, follow-up timepoints (e.g., Week 24 and Week 48) were defined relative to ART initiation. In contrast, in our prospective cohort, baseline (Day 0) coincided with ART initiation, as all participants started treatment at their first clinic visit. Thus, retention estimates in the intervention group include all enrolled participants from their first contact with care, whereas in the control group only those who initiated ART contributed to retention denominators.

Because data for historical controls were abstracted retrospectively from treating physicians rather than from a centralized registry, individuals who linked to care but never initiated ART, or who started ART and did not return thereafter, were under-ascertained. As a result, retention in controls may be overestimated, introducing a conservative bias against the intervention cohort. Nevertheless, this discrepancy highlights a key advantage of rapid initiation: the elimination of pre-ART attrition, a major source of loss to care among PWID.
Among historical controls, the median time (25th–75th percentile) from HIV diagnosis to ART initiation was 78 (28–297) days, totaling 60,061 community pre-ART viremia-days. In the rapid-initiation cohort, baseline coincided with same-day ART, yielding 0 pre-ART viremia-days by design. This extended diagnosis-to-ART interval represents a period of uncontrolled viremia during which syringe sharing carries a high risk of onward transmission, whereas once viral suppression is achieved the risk of sexual transmission is effectively zero (U=U). By eliminating this interval, the integrated rapid-start model would be expected to reduce community exposure to infectious blood and contribute to population-level prevention benefits among PWID.

Supplementary Figure S3. Visit Adherence Over Time

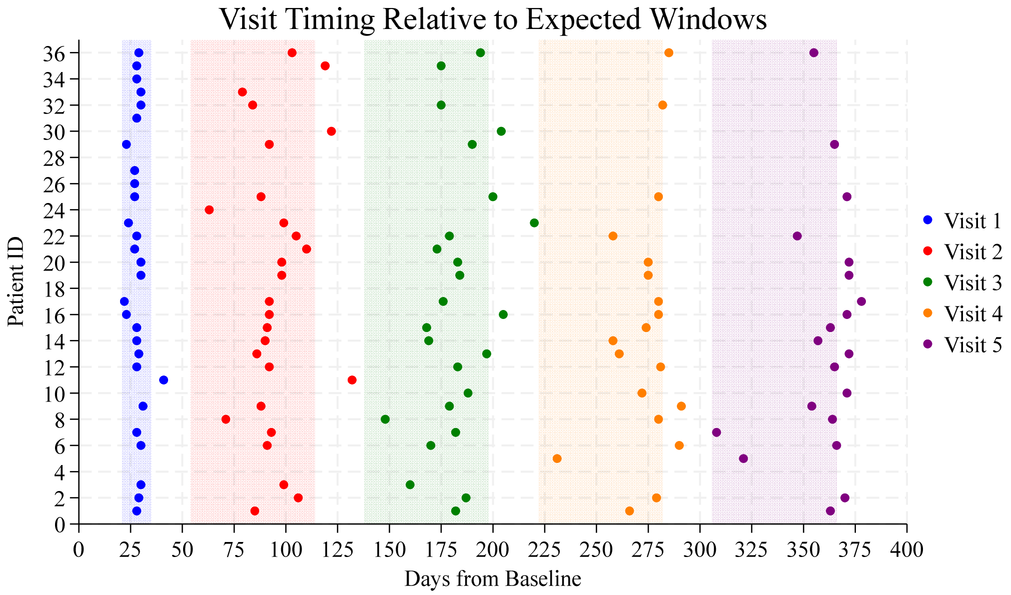

Distribution of days from scheduled visits at Weeks 4, 12, 24, 36, and 48; medians and interquartile ranges illustrate increasing variability over time among retained participants.

Supplementary Figure S4. Visit completion patterns from baseline through Week 48

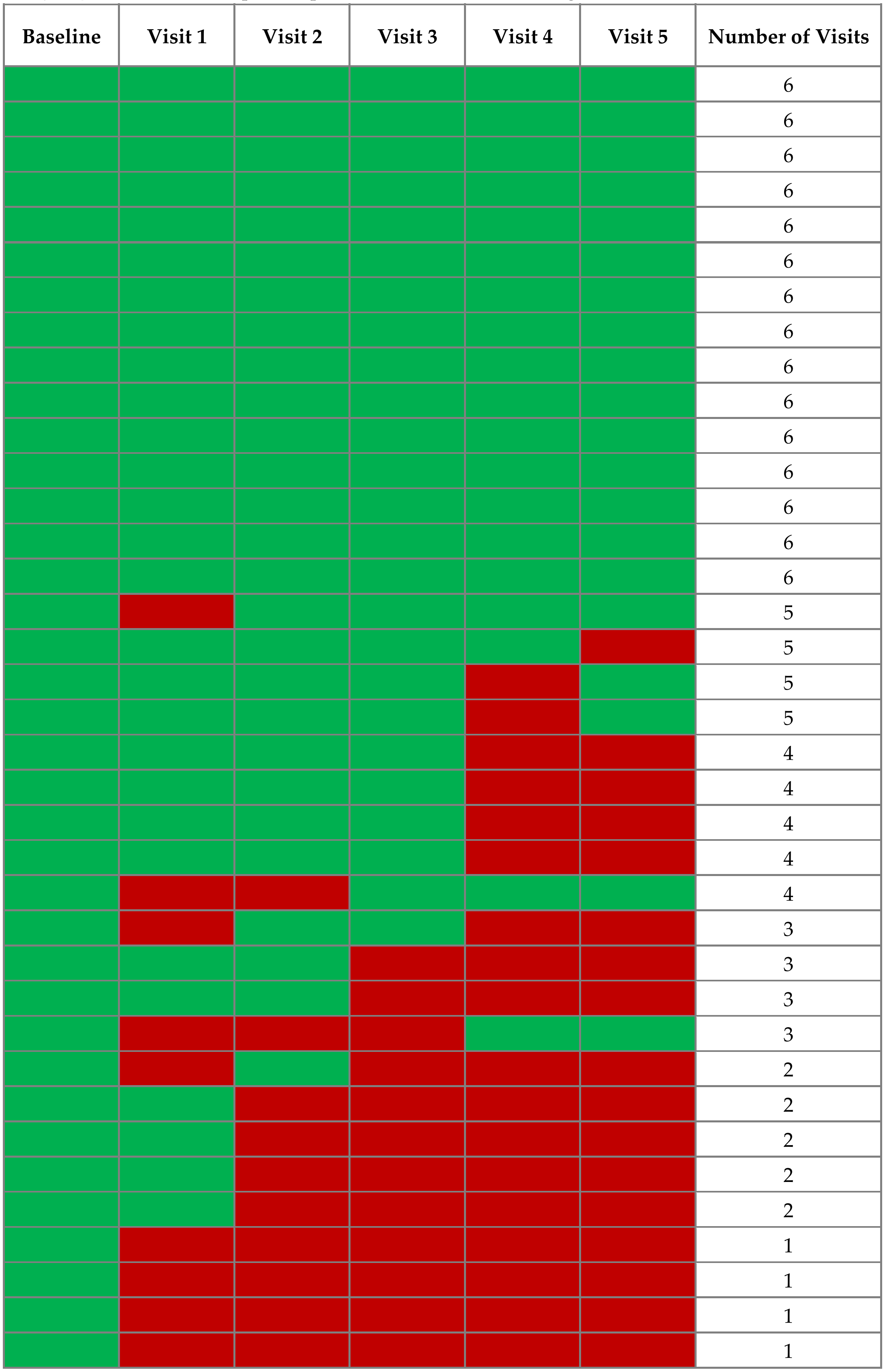

Each row represents one participant; green = completed visit, red = missed. Columns correspond to Baseline and Weeks 4, 12, 24, 36, and 48.

Supplementary Figure S5. Individual Viral Load Trajectories

Observed HIV-1 RNA values over time among participants with at least one follow-up assessment.

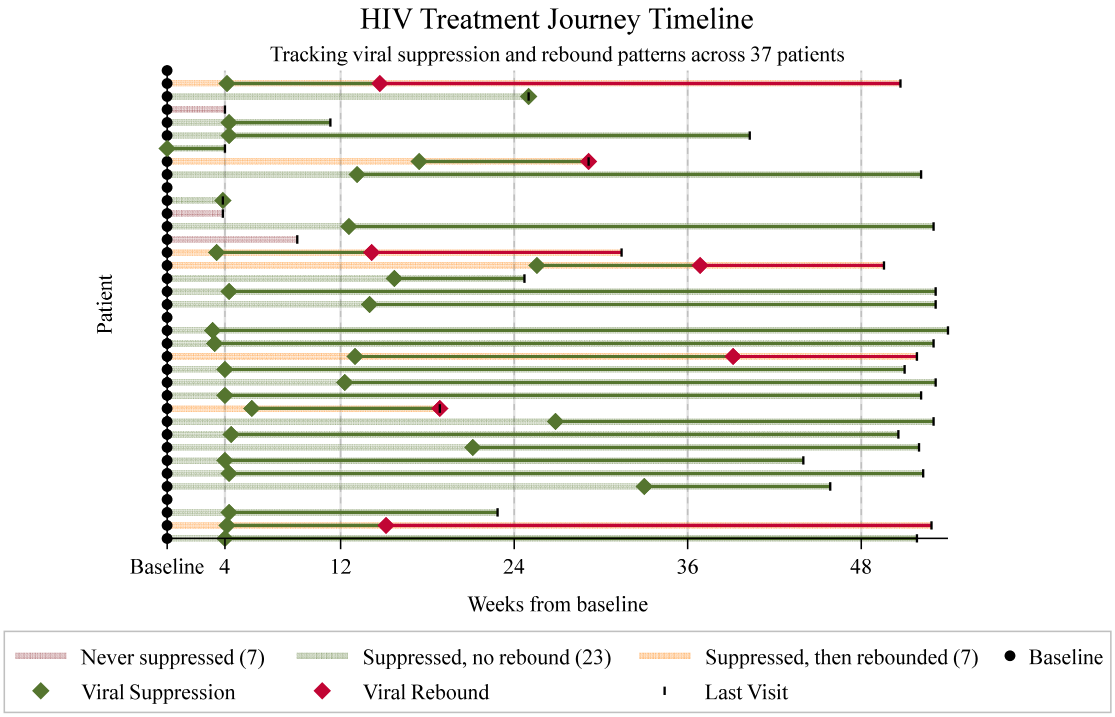

Supplement: Supplementary file 1 [file microorganisms-13-02697-s001.zip › microorganisms-4003114-supplementary.pdf]
